# Supplementary material for: Are carbon emissions trading and green financial instruments synergistic? -Comprehensive quantitative research based on content analysis
Source: PLoS One. 2024 Mar 7;19(3):e0298601. doi: 10.1371/journal.pone.0298601 (PMC10919601; doi:10.1371/journal.pone.0298601)
Supplement: S1 Data — (ZIP) [file pone.0298601.s001.zip › Data/地级市Z_F工作报告.zip!/╣π╢1⁄2/╤⌠╜¡╩╨/└┤╫╘╣1⁄2╓┌║┼í╛┬φ┐╦╩2╛▌═°í┐_2005.docx]

各位代表：

　　我代表市人民政府向大会作政府工作报告，请予审议，并请政协各位委员和其他列席人员提出意见。

2004年工作回顾

　　2004年是本届政府任期的第一年。一年来，在中共广东省委、省人民政府和中共阳江市委的正确领导下，在市人大及其常委会和市政协的监督支持下，市政府坚持以邓小平理论和“三个代表”重要思想为指导，认真贯彻中央和省的工作部署，围绕市第四次党代会和市四届人大一次会议确定的目标任务，牢固树立和落实科学发展观，以发展为第一要务，以增强综合实力和城市竞争力为核心，在全力推进各项工作的同时，重点抓好调整完善发展思路、优化发展环境、解决群众困难等工作。结合阳江发展实际，把工作重点从发展轻工、旅游业调整到打造广东电力能源基地、发展临港工业、建设面向西南的物流配送基地上来，加大招商引资力度，努力做大工业，强化经济发展动力；加快推进交通基础设施和电源电网建设，夯实发展基础；整顿机关作风，规范收费行为，整治社会治安，努力营造良好发展环境；坚持以人为本，着力解决农村征地、城镇房屋拆迁、国企改革等遗留的关系人民群众生产生活困难的重点问题，确保社会稳定，促进国民经济持续快速健康发展和社会各项事业全面进步，推动三个文明建设协调发展。在全市人民的共同努力下，市四届人大一次会议确定的各项任务顺利完成。

　　——经济发展跃上新台阶，质量效益同步提高

　　经济总量不断壮大。全市完成生产总值273.9亿元，增长12.6%，其中第一、二、三产业分别增长5.5%、21.1%和10.8%。经济总量在全省排名由上年的第16位上升到第15位。三大产业比例由上年的32.9：34.9：32.2调整为30.4：38.0：31.6，二、三产业比重全面超过第一产业。

　　农业农村经济有新发展。全年实现农业总产值128亿元，增长5.6%。认真落实中央1号文件和扶持粮食生产的各项政策，粮食生产在40年未遇的大旱情况下保持较好收成。全年粮食种植面积228万亩，增加5.2万亩；粮食总产量76万吨，增长3%。农业和农村经济结构不断优化，优质水稻、水果、蔬菜、南药种植规模进一步扩大，农村二、三产业有新的发展。积极扶持发展40家农业龙头企业，带动农户19.5万户，户均增收1865元。农产品加工和流通水平提高，家具、荔枝、藤类工艺品等打入欧洲市场。农业标准化和农业现代化示范区建设稳步推进，农产品质量安全工作得到加强。重视农田水利基本建设，水利主干渠清淤工作取得明显成效。林业工作取得较好成绩，森林资源得到有效保护。高致病性禽流感疫情得到及时有效控制，人民生命财产得到有效保护，其它重大动植物疫病的监控和防治得到加强。积极采取措施减轻农民负担，取消、免收或降低涉农收费18项，减负1748万元。

　　新型工业化进程加快。深入开展工业发展年活动，扶持工业发展的政策措施进一步落实。全年新上工业项目516个，总投资58.4亿元。加强高新区和各类工业园区建设，大力发展临港工业。日本长崎（阳江）科技园、同济大学天虹（阳江）科技园等45个项目落户高新区。产业结构进一步优化升级，钢铁、汽配等重工业发展加快，在规模以上工业中，重工业增速比轻工业高22.2个百分点。技术改造和创新力度加大，积极运用全省工业产业竞争力研究成果，推进重点行业和骨干企业技术进步。全年向省上报技术创新和技术改造重点项目58项，总投资11.3亿元。大力实施名牌带动战略，全市新增中国驰名商标1 件，中国名牌产品1个，广东省著名商标11件，广东省名牌产品6个。举办第三届中国（阳江）国际刀剪博览会，构筑五金刀剪全球交易平台，推动五金刀剪产业发展提高。全年五金刀剪产业完成产值62亿元，增长17%。继2001年阳江获得“中国刀剪之都”荣誉后，去年又被命名为“中国五金制品（刀剪工具）出口基地”。全年实现工业总产值280亿元，增长20%。工业对经济增长的贡献率为46.3%，提高9个百分点。

　　第三产业稳步发展。连锁经营、物流配送、批发市场、电子商务等现代流通业发展加快，全年新增规模以上连锁经营店、仓储式商场、超市等商业网点10个，经营面积6.5万平方米。文化、体育、餐饮、医疗保健等服务业稳步发展，汽车、住房、通讯等消费需求畅旺。全年完成社会消费品零售总额109.8亿元，增长13%。第三产业实现增加值86.6亿元，增长10.8%。

　　落实宏观调控政策取得初步成效。坚持有保有压的原则，优化投资结构，农业、基础设施、支柱产业和社会公共事业投资进一步加强。全社会固定资产投资完成65.5亿元，增长13.9%。大力整顿和规范土地市场，加强土地集约利用，保障重点项目建设用地。全市清理违法用地1095.1公顷，收回闲置土地7.9公顷。大力推进经济结构调整和经济增长方式转变，经济增长质量和效益稳步提高。地方财政一般预算收入7.4亿元，增长12.6%（按可比口径计算）。金融机构人民币各项存款余额218.3亿元，贷款余额107亿元，分别比年初增长13.3%和6.9%。规模以上工业企业经济效益综合指数113.3，提高1点。加强经济运行调节，煤电油运等的制约得到有效缓解。市场物价基本保持稳定，居民消费价格总水平上升2.8%。

　　——特色经济活力增强，县域经济取得新发展

　　民营经济实力壮大。进一步落实扶持民营经济发展的政策措施，民营经济发展势头强劲。全年净增民营企业、个体工商户3267户，其中工业企业、个体工业户777户。民营经济实现增加值158亿元，拉动经济增长8.1个百分点。丰源粮油等一批民营企业不断增资扩产，规模实力明显提高。全年民营工业产值218.3亿元，增长20.4%，占全市工业总产值的78%。有进出口经营权的私营企业出口总额3亿美元，增长65.1%，占全市出口总额的35.9%。

　　海洋渔业稳步增长。远洋渔业有效拓展，养殖业规模不断扩大。平冈农场获“全国无公害养殖基地”称号。水产品加工水平提高，新建了海鑫、圣海等一批水产品加工企业。水产企业国际认证工作得到加强，水产品出口总值居全省第三位。粤西水产品综合批发市场建成开业。种苗生产不断发展，全市建成规模较大的水产种苗场145个。渔港基础设施建设加快，闸坡国家级中心渔港建设稳步推进，沙扒渔港被定为国家一级渔港。成功举办第二届南海（阳江）开渔节。设立了南鹏岛水产自然保护区。全市水产品总量88.8万吨，增长2.9%；渔业总产值45.3亿元，增长6.3%。水产品总量和人均占有量连续6年居全省首位。

　　旅游业持续发展。打好海陵岛大角湾AAAA级景区和阳春凌霄岩国家地质公园两个“国字号”品牌，整合旅游资源，打造旅游精品线路，促进旅游区域联动发展。加强景区景点建设，重点推进雪流湾旅游综合开发、东方银滩欢乐广场、月亮湾健康旅游度假中心等项目的建设。举办了第五届旅游文化节和首届阳江美食节。全年接待游客366.8万人次，旅游总收入17.7亿元，分别增长32.1%和30.7%。

　　县域经济不断壮大。制定加快发展县域经济的政策措施，调动县级政府发展经济的积极性。各县（市、区）在招商引资、发展工业、培育财源、转移农村劳动力、增加农民收入等方面均取得明显成效，经济实力日益增强，综合竞争力不断提升。

　　——招商引资工作有新成效，对外开放水平有新提升

　　阳港澳合作更加密切。以泛珠三角区域合作战略的实施为契机，加强与港澳地区的交流与合作。积极组织企业参加粤港澳CEPA合作交流会、粤港经济技术合作交流会、第九届澳门国际贸易投资展览会、泛珠三角经贸合作交流会等经贸活动。成功举办阳港企业经贸合作交流会，确定了一批合作项目。阳港澳的合作领域不断扩大，合作水平不断提升。

　　吸收外资成效显著。制定和实施《关于进一步加强招商引资工作的决定》和《阳江市招商引资奖励办法》，优化投资环境，加强宣传推介，创新引资方式，成功引进中国保利集团、广东粤电集团等企业的投资，利用外资工作取得新成绩。全年合同利用外资1.8亿美元，增长29.7%；实际利用外资5750万美元（按新口径统计），完成省下达我市任务的113%。

　　外贸出口实现较大增长。深化外贸体制改革，调整优化出口商品和出口市场结构，大力扶持中小企业出口，积极帮助民营企业开展自营进出口，外贸出口持续增长。全年完成外贸进出口总额10.7亿美元，增长33%。其中出口总额8.4亿美元，增长22%。

　　——重点项目建设步伐加快，基础设施不断完善

　　重点项目建设取得新突破。阳江核电站获得国家批准立项，前期工程稳步推进。阳江抽水蓄能电站、阳西燃煤电厂、海陵岛风电场的前期工作进展顺利。广东海上丝绸之路博物馆正式奠基。阳茂高速公路阳江段、省道113线阳春南段一期工程、阳阳铁路、阳江港3万吨级粮食码头已建成使用。阳江港至云浮高速公路建设项目前期工作全面启动。珠三角成品油管道阳江段完成焊接工程。城乡防灾减灾工程建设加快，市区1.8公里防洪工程顺利完成，阳春城市防洪工程全面铺开。完善了白沙、永宁等4个110千伏输变电工程建设，农村电网进一步改善。电信扩建扩容工程有序推进。20个重点项目完成投资20.5亿元，提前完成预期目标。

　　城市建设呈现新面貌。城市基础设施不断完善，完成了市人民广场、文化广场和创业北路一期、新江北路续建工程。推进了安宁路中段、马南中路、滨江东路、体育北路、新江东路等市政道路的建设。建设了市垃圾渗滤液处理厂和医疗废物处理中心。启动了城南新区的规划和各县（市）污水处理厂的建设。城市管理水平不断提高，结合开展创建国家卫生城市、园林城市和环保模范城市活动，加强城市管理和环境监测，搞好城市净化绿化美化亮化，市容市貌有了较大改观。城市活力不断增强，成功引进碧桂园等知名企业参与城市开发建设。建筑业、房地产业健康发展，建成一批新型住宅小区。湖湾华庭小区被认定为 “广东省绿色住区”。县级城市不断拓展，中心镇的规划建设加快。

　　——各项改革继续深化，经济体制逐步完善

　　行政审批制度改革继续推进，取消、减少行政许可事项190项。国有企业改革步伐加快，市龙达环保设备厂、市工业开发总公司等国有企业的改制工作顺利完成，阳江港开发总公司、市物资总公司、市城建集团、八甲电厂等国有企业的改制工作全面启动。农村税费改革稳妥推进，全市减免农业税1533.5万元，人均减负7.9元。市县机构改革和撤村并镇工作全面完成。投资体制和粮食流通体制改革有序进行。整顿和规范市场经济秩序工作深入开展，传销和变相传销等违法犯罪活动得到遏制，市场经济秩序进一步好转。

　　——科教人才工作不断加强，文化名城建设稳步推进

　　实施科技创新9项计划和各类科技计划项目，抓好高新技术和先进适用技术的推广应用，全年获国家、省批准立项的科技计划项目36个，新增省级高新技术企业14家、省级民营科技企业12家。以企业为主体的技术创新体系建设加快，新建市级以上工程技术研究中心11家。产学研结合有新突破，建立了华南理工大学阳江工业技术研究院。知识产权保护工作得到加强，全市专利申请量499件，增长35.2%，连续7年保持粤西地区领先地位。教育工作得到加强。高中阶段教育发展加快，职业教育取得新成绩，高等教育不断扩大。全市4所学校晋升为省一级学校，3 所学校被定为省首批示范中职学校，阳江职业技术学院在校生超过5000人。同心中学一期工程和第三批37所老区农村小学危房改造基本完成。教育信息化建设取得新成绩，被教育部初步确定为全国信息技术教育示范区。完善人才工作机制和配套措施。全年举办15期人才交流会，为企业和单位引进了一批优秀人才。粤西区域性人才市场建设进展顺利。文化事业发展加快。图书馆、博物馆、科技馆和群众艺术馆建设有序进行，群众文化活动活跃。文化市场健康发展，文化经济和文化产业发展加快。现代公民教育全面启动，文明社区、文明村镇、文明行业等群众性文明创建活动稳步推进。

　　——民主法制建设深入推进，社会保持和谐稳定

　　民主法制建设不断加强。坚持向人大及其常委会报告工作、向政协通报情况制度，自觉接受法律监督和民主监督。加强与各民主党派、工商联和工会、共青团、妇联等组织的联系，广泛听取意见。认真做好议案、建议和提案办理工作，全年办理人大议案3件、代表建议119件，政协建议案1件、委员提案88件。政务、厂务、村务公开深入推进。城乡基层组织建设和企事业单位民主管理不断加强，基层民主进一步扩大。普法宣传教育广泛开展，群众法律意识不断增强。健全信访工作制度，落实信访工作责任制，全市信访总量8年来首次下降。

　　依法行政工作全面推进。认真贯彻实施行政许可法和《全面推进依法行政实施纲要》，重新修订《阳江市人民政府工作规则》，规范政府行为，强化行政行为监督。加强党风廉政建设和审计监督工作，市政府向社会公开作出廉政承诺并认真履行。坚决纠正损害群众利益的不正之风。加大改革力度，健全制度和机制，从源头上预防和治理腐败。

社会治安综合治理初见成效。加强社会治安防控体系建设，切实加强城乡社会治安综合治理。深入开展严打整治斗争，重拳打击“双抢”、“两盗”犯罪活动，严厉打击“六合彩”等赌博活动，大力扫除“黄赌毒”等社会丑恶现象，深入开展对“法轮功”邪教组织的斗争，社会治安和秩序逐步好转。

　　安全生产形势好转。落实安全生产责任制，推行量化目标管理，实行隐患整改指令书跟踪落实制度，加强对重大危险源的监控和专项整治，各类安全事故明显减少，较好地完成安全生产控制目标任务。

　　——社会各项事业全面进步，群众生产生活条件有效改善

　　就业社保工作不断加强。全市新增城镇就业岗位3.4万个，下岗失业人员实现再就业9525人，城镇登记失业率为3.26%。积极推进社会保障体系配套改革，不断完善城乡居民最低生活保障制度和农村五保供养制度，加强社保基金管理，社会保险实现了市级统筹，覆盖面进一步扩大，全市参加基本养老、医疗保险人数达21.5万和11.7万人，分别增长2%和33.5%。

　　群众生产生活条件逐步改善。积极实施十项民心工程，突出抓好城镇廉租住房、经济适用住房建设和农村贫困户泥砖住房改造以及饮水工程建设。全市建设廉租住房和经济适用住房236套，面积1.9万平方米；改造泥砖住房9828户，面积71.2万平方米；完成农村饮水工程项目18个，城乡居民住房难、饮水难问题得到初步缓解。完成542.6公里镇到行政村公路硬底化工程。大力清理拖欠农民征地补偿款和建设领域拖欠工程款、农民工工资，全市清欠农民工工资2666.8万元，完成清欠任务的98%；全部兑付157宗征地补偿款共24879万元。城乡居民生活水平不断提高，城镇在岗职工年人均工资收入12600元，增长17.5%；农村居民人均纯收入4420元，增长7.8%。加强环境资源保护和生态建设，城乡居民生活环境进一步改善。

　　社会各项事业持续发展。加强公共卫生三大体系建设。市疾控中心大楼和市人民医院感染性疾病中心大楼已完成主体工程。稳步推进新型农村合作医疗制度，农村合作医疗覆盖面为43.6%，提高10.1个百分点。艾滋病等重大疾病的预防控制工作取得新进展。无偿献血工作连续三次荣获“全国无偿献血先进城市”称号。广泛开展全民健身运动，不断提高竞技体育水平。人口和计划生育工作取得新成绩。在全国地级市率先实行农村部分计划生育家庭奖励制度。全年人口自然增长率为6.7‰。有序推进经济普查工作。国防动员、人民武装和双拥共建工作不断加强，连续四次被评为“省双拥模范城”。统计、民政、外事、侨务、民族、宗教、妇女儿童、人防、打私、气象、地震、档案、市志、爱卫等各项工作都有新发展。

　　各位代表，过去一年，我市国民经济和社会发展取得的成绩，是省委、省政府和市委正确领导的结果，是各级人民代表大会及其常委会和人民政协监督支持的结果，是全市人民认真贯彻 “三个代表”重要思想，全面落实科学发展观，团结奋斗，开拓进取的结果，是各民主党派、社会各界人士以及广大投资者、港澳台同胞、海外侨胞关心支持的结果。在此，我代表市人民政府，向全市广大工人、农民、知识分子、干部职工、驻军部队、武警官兵、人民警察和各界人士表示崇高的敬意！向为全市现代化建设作出贡献的广大劳动者、建设者和长期关心支持阳江经济社会发展的港澳台同胞、海外乡亲、国际友人表示衷心的感谢！

　　与此同时，我们还必须清醒地看到，我市起步晚、基础差、底子薄，经济社会发展中存在的一些困难和问题还远未解决好。面对新的形势和任务，还有很多新的问题有待我们去面对和解决。主要是：经济总量小，结构不合理；科技创新能力较弱，产业竞争力不强；投资不足，储备项目少，发展后劲乏力；税源不足，财政收支平衡压力较大；社会治安压力大，建设和谐社会的任务艰巨；部分干部发展意识淡薄，工作责任感不强；政府机关作风建设还存在薄弱环节等。对这些问题，我们将采取切实措施认真加以解决。

2005年工作总体要求和目标任务

　　2005年是全面实现“十五”计划目标、衔接“十一五”发展的关键一年。当前，世界经济复苏，全球产业结构调整转移步伐加快，随着我国即将全面履行入世承诺，市场经济国家地位逐步得到广泛承认，必将为我们参与国际分工、承接产业转移、优化产业结构带来良好机遇；区域经济一体化趋势，东盟10+1区域经济合作，CEPA的实施和泛珠三角区域合作的推进，有利于我市发挥错位竞争优势，推动外向型经济发展。国内政治社会稳定，经济发展周期处于上升期，经济保持平稳较快发展，有利于我市跨越式发展战略的实施。我市产业发展、基础设施建设和区域合作取得新突破，经济自主增长和承接产业转移能力不断增强，而且阳江核电站等重大项目将陆续上马建设，带动效应逐步显现，发展空间进一步拓展，同时经过多年的实践，全市上下形成想发展、致力于科学发展的共识，具备了跨越式发展的条件。但是，我们必须看到，影响我市经济社会发展的不稳定、不确定因素也不少，加上省内兄弟市发展势头迅猛，我市加快发展的竞争压力增大。对此，我们必须保持清醒的头脑，树立强烈的机遇意识和忧患意识，紧紧抓住并切实用好重要战略机遇期，统筹谋划发展新思路、新途径，促进经济社会全面协调可持续发展。

　　2005年政府工作的总体要求是：高举邓小平理论和“三个代表”重要思想伟大旗帜，认真贯彻党的十六大、十六届三中、四中全会和中央经济工作会议以及胡锦涛总书记两次视察广东重要讲话精神，按照省委九届六次全会和市委四届三次全会的工作部署，坚持以科学发展观统领经济社会发展全局，以发展为第一要务，以加快建设工业新城、生态绿城、旅游美城、文化名城为总目标，以工业化为主导，以招商引资为突破口，抢抓机遇，深化改革，扩大开放，大力发展临港工业，打造广东电力能源基地，建设面向西南的物流配送基地，推动经济快速持续健康发展；坚持以人为本，加强依法治市，注重社会公平，改善群众生活，维护社会稳定，构建和谐阳江，确保 “十五”计划目标顺利实现。

　　2005年我市经济社会发展的主要预期目标是：生产总值增长12%，社会消费品零售总额增长13%，固定资产投资增长15%，外贸出口增长6%，实际吸收外商直接投资增长15%，居民消费价格总水平涨幅控制在3%左右，地方财政一般预算收入增长10%，城镇登记失业率控制在3.5%以内，人口自然增长率控制在7‰以下，在岗职工年均工资实际增长6%，农村居民人均纯收入实际增长4%。

　　按照上述总体要求，做好今年工作，我们要落实省委、省政府对今年工作的总体要求，坚持抓落实、重提高、促发展，树立全局意识、使命意识、创新意识，突出做到四个必须：

　　一是必须牢固树立加快发展思想。我市目前仍属欠发达地区，经济基础差、底子薄、总量小，不论是经济整体素质，还是发展速度，与全省特别是珠三角等发达地区相比都有较大差距。我们必须增强危机感、紧迫感和责任感，牢固树立抢抓机遇、加快发展思想，以昂扬向上的精神状态，敢想敢拼的进取精神，求真务实的工作作风，充分发挥比较优势，抢抓机遇，急起直追，努力实现跨越发展。

　　二是必须进一步改善投资环境。阳江建市17年来，在册的外资企业只有300家，去年年检的仅有178家。这与我市处在沿海地区的位置极不相称。其中重要原因是我市的投资环境没有得到根本改善和优化，主要表现在：电源电网建设滞后，部分干部群众发展意识淡薄，一些部门服务意识不强，行政行为和行政收费不规范，社会治安形势比较严峻等。全市上下必须形成共识，抓住泛珠三角区域合作带来的新一轮经贸合作与投资热潮机遇，真正把优化投资环境作为发展的生命线来抓，在致力改善交通基础设施和电源电网建设的同时，下大决心，拿硬措施整顿机关作风，清理行政收费，整治社会治安，力争投资环境短期内有明显改善。

　　三是必须进一步调动广大干部群众的积极性。阳江人民的开放意识和营商意识历来较强，我们必须充分挖掘和弘扬阳江历史上的重商传统，进一步调动广大干部群众的积极性，鼓励他们建言献计，利用亲情、友情、乡情等关系，大力开展招商引资，为全市发展争作贡献，形成万众一心、共谋发展的强大合力。

　　四是必须重视解决群众关心的热点难点问题。群众利益无小事。我们必须坚持以人为本，时刻把工作的出发点和立足点放在维护人民群众根本利益上，重视解决关系人民群众生产生活的热点难点问题，坚决纠正损害群众利益行为，切实把人民群众的根本利益实现好、维护好、发展好，努力营造和谐稳定的社会环境。

　　为实现今年经济社会发展预期目标，必须做好以下工作：

　　一、大力推进新型工业化，强化工业主导地位

　　大力发展电力工业。认真做好服务和协调工作，配合抓好阳江核电站、阳江抽水蓄能电站、阳西燃煤电厂、海陵岛风力发电场四大电力项目建设，加快发展与电力相关的配套产业，带动工业跨越式发展。

　　加快发展临港工业。充分发挥临港优势，推动一批石化、钢铁、海洋生物、农副产品深加工等临港工业的发展。重点利用毗邻茂名、珠海等石化基地的优势，大力发展石化中下游产品加工项目，加快油气化工仓储设施建设。抓好广东丰源粮油工业有限公司二期工程、广州造纸厂林纸基地、阳江川崎特殊钢实业有限公司、阳江市德恩富科技有限公司光碟生产等项目的跟踪落实和服务工作。加快推进金山民营科技园标准厂房示范区、同济大学天虹(阳江)科技园、日本长崎(阳江)科技园的建设，推动临港工业新发展。

　　积极发展装备制造业。制定装备制造业发展规划及实施方案。坚持引进先进技术和自主创新相结合、政策扶持和市场竞争相结合，扶持发展一批装备制造业企业。重点做大做强阳江宝马利汽车空调有限公司、阳春轴承股份有限公司、阳西电梯配件有限公司等企业，推动汽配及装备工业加快发展，逐步把我市建设成为装备制造业基地。

　　改造提升传统产业。坚持推进新型工业化与建设制造业基地相结合，广泛运用高新技术和先进适用技术改造提升传统产业，重点加强五金刀剪、食品加工、医药化工、家具编织等重点行业和骨干企业技术创新，推动传统特色产业优化升级。鼓励企业应用信息技术提升竞争力。引导社会资金参与企业技术改造。

　　二、着力解决“三农”问题，保持农业农村稳定发展

　　提高粮食综合生产能力。全面落实种粮补贴、农机补贴、粮食最低收购价、农资价格监管、地力培育等措施。加强对基本农田的保护和建设，严格控制非农建设占用基本农田。加强农业基础设施建设，加快中低产田改造，提高粮田产出水平。增加对粮食优势区域投入，实施优质稻产业工程。鼓励种粮大户发展规模经营，开发优质粮食品牌，推进粮食产业化经营。

　　加快农业产业化进程。在确保粮食安全的前提下，科学规划农业区域布局，整合农业项目、资金和生产资源，因地制宜，发展具有亚热带特色的果、菜、花、茶、药等高值高效作物，鼓励发展“一镇一业”、“一村一品”，培育发展区域特色产品和优势产业，推动大宗农产品向优势产区集中。加快农产品加工体系建设。发展农民专业合作经济组织，建设、扶旺一批农产品专业批发市场。加大农业龙头企业发展的扶持力度，努力新增一批市级以上农业龙头企业。加快农业科技创新，抓好优质农作物品种的选育、引进和推广，加强优质种子（种苗）基地建设，扩大主要农作物新品种覆盖面。加快农业先进机械、设施的应用。实施科技兴林工程，推进林业产业化。

　　完善农产品质量安全体系。加强农产品质量安全监督检测体系建设，逐步实行农产品市场准入制度。加快建设农产品质量安全监测信息网。认真抓好农业示范区建设，努力新增一批省、市级农业标准化示范区。重点抓好阳春马水桔、春湾蔬菜、双　沙姜，阳东双肩玉荷包荔枝，阳西上洋西瓜等农产品的标准化生产。加强动植物疫情监测，建立和完善疫情报告、预警机制。

　　减轻农民负担。按照省的部署，全面免征农业税。落实减轻农民负担行政首长负责制和部门责任制，深化村务公开工作，健全各种监督管理机制，抓好各种涉农收费的专项治理，严肃查处加重农民负担的违法违纪行为。逐步推行村级会计委派制、选聘制或代理制。加强农村集体经济审计监督，规范集体资产和财务管理，积极稳妥化解农村不良债务。

　　三、继续推动外向带动战略，大力加强招商引资

　　优化投资环境，扩大外资规模。简化投资审批程序，健全投资服务体系，提高办事效率，优化服务质量。大力整治涉企收费，严厉打击损害企业利益行为，制定《关于降低收费门槛，促进招商引资的决定》。完善优惠政策，落实奖励措施，调动全社会招商引资积极性。创新招商引资方式，以龙头项目带动产业招商，继续推行驻点招商、网上招商、委托代理招商、企业自主招商。进一步密切与港澳和珠三角等发达地区的联系，共建“产业转移园区”，承接一批产业转移项目。促进现有外资企业增资扩产。抓好签约项目和在谈项目的跟踪落实工作。

　　转变外贸增长方式，保持出口稳定增长。推动贸易方式和市场多元化，巩固港澳、东盟、欧美日等传统市场，大力开拓非洲、南美、俄罗斯等新兴市场。实施科技兴贸，优化出口商品结构，提高出口商品附加值。赋予更多符合条件的民营企业进出口经营权，大力扶持本地产品特别是有自主知识产权和自主品牌产品出口。鼓励和扶持发展加工贸易。

　　四、做大做强特色产业，大力发展县域经济

　　提升五金刀剪产业。加快五金刀剪技术创新中心、产品检测中心建设，构筑产业技术创新平台和服务体系，提高产业自主创新能力，增加自主品牌，推动产业转型升级。强化市五金刀剪行业协会的作用，抓好行业管理、质量监测和市场开拓等工作，提高产业集群发展水平。完善“中国刀具中心”网站，进一步打响“中国刀剪之都”品牌。

　　优化海洋产业。办好第三届南海（阳江）开渔节。继续推进虾、牡蛎、罗非鱼等优势品种的规模化养殖和产业化经营，提高水产养殖业水平。突出抓好水产品精深加工，加强产品质量管理，打造水产品牌。抓住欧盟对我国水产品出口全面解禁的有利时机，鼓励水产企业开展国际标准认证，开拓国际市场。加强闸坡国家中心渔港和沙扒国家一级渔港建设，抓好东平国家中心渔港申报工作。加强水产种苗基地建设，鼓励企业创建国家级和省级良种场。加快发展以海洋化工、生物制药、保健品、化妆品等为重点的海洋加工业、滨海旅游业和海洋运输业，全面发展综合型海洋经济。

　　做旺旅游业。完善重点景区景点建设，加快推进广东海上丝绸之路博物馆、雪流湾旅游综合开发、东方银滩欢乐广场、月亮湾健康旅游度假中心、鹅凰嶂自然保护区旅游综合开发等项目建设。积极吸引民资外资参与景区景点和旅游配套设施建设，开发一批具有地方特色和文化内涵的旅游商品、纪念品，建设一批高等级宾馆和旅游商场，进一步完善食、住、行、游、购、娱六大要素。积极参与泛珠三角区域旅游合作，推动区域旅游联动发展。发挥旅游协会作用，加强行业自律和管理。办好第六届旅游文化节，加大旅游推介促销力度，提高阳江旅游的知名度和美誉度。

　　壮大南药加工业。把南药生产加工作为区域特色经济的新亮点来抓，在进一步抓好春砂仁、穿心莲、板兰根、霍香等南药的规模化生产和产业化经营的同时，加强对阳江制药厂、粤西绿色工业有限公司、柯瑞生物工程医药公司、阳春一片天制药集团等制药企业的扶持和引导，培育龙头企业，带动全市南药加工业的更大发展。

　　五、加快发展现代物流业，大力培育发展第三产业

　　以发展现代物流业为突破口，推动第三产业加快发展。加快推进阳江港保税物流中心、阳江烟草配送中心、粤西粮油批发配送中心的建设，大力发展临港物流业。抓好商业网点规划，结合产业和资源优势，推进五金刀剪、农产品、海产品、汽配、建筑装饰材料等专业特色批发市场规划建设，加快第一市场、岭东市场等农贸市场的改造建设步伐，培育发展一批物流基地。抓好流通业的改革和发展，大力发展连锁经营、物流配送、中高级批发市场等新的流通业态。扩大服务业对外开放，加快商务、物流、会展等服务业的发展，推动新型服务业快速成长。

　　六、推进重大项目建设，保持投资有效增长

　　加快电源电网建设。完善中长期电源电网建设规划。加快推进四大电源项目前期工程建设。抓好石湾、岗美、合山、东平4个110千伏和漠南220千伏、蝶岭500千伏输变电工程建设，着力解决输变电站和电网“卡脖子”问题。继续推进城网、农网改造。

推进交通基础设施建设。加快推进阳江港至云浮高速公路建设的前期工作。抓好阳江港3.5万吨级煤码头、5万吨级油气码头建设和5万吨级航道疏浚工作。大力推进阳茂高速公路至阳西县城、省道113线阳春南段二期、省道369线阳春段、溪头至沙扒等公路项目建设。完成530公里镇到行政村公路硬底化工程。

　　抓好城市开发建设。做好市区整体规划修编工作。加快推进城南新区、核电站生活科研后勤基地的规划建设和阳东燕山湖综合开发。抓好滨江东路、石湾北路、西平北路、马南中路、体育路北段、安宁路中段等在建项目和甘泉路建设，加快世纪公园建设步伐。启动东风四路、新江南路、高凉路、创业北路二期工程的改造建设。抓好市第一净水厂扩建和管网改造，加快建设各县（市、区）污水处理厂。继续完善市区管道天燃气管网建设。全面推进创建国家卫生城市、园林城市和环保模范城市工作，进一步搞好城市的净化绿化亮化美化。抓好县级城市建设，大力推进中心镇的规划建设，加快城镇化步伐。

　　加强城乡水利防灾减灾工程建设。重点推进市区和阳春、阳西的城市防洪工程建设，加快大中型水库和江海堤围除险加固达标建设步伐，抓好农田水利设施的综合治理。

　　七、加大促产培财力度，强化财政金融工作

　　积极培育财源。加大对财政贡献大、符合国家产业政策的高新技术企业和重点企业的扶持力度，引导现有企业增资扩产，发展一批龙头骨干企业。选准和发展特色支柱产业，培育特色经济，壮大园区经济，提升劳务经济，做大做强县域经济，培育财源，增加财政收入。

加强税收征管。巩固协税护税网络建设，提高公民纳税意识。坚持依法治税，切实加强税收收入征管，规范非税收入和国有资产收益收入征管。加强重点地区和税源企业的监控，严厉打击偷抗逃骗税行为，堵塞漏洞，确保财税增长与经济增长协调发展。

　　规范财政管理。建立本级财政支出的有效监督机制，加强预算约束，严格控制预算追加，确保财政对 “三农”、教育、科技、卫生、社保、环保等支出需要。强化收支“两条线”管理，完善部门预算，全面铺开国库集中支付制度，实行市直公务员岗位津贴统一发放。加大对政府部门预算执行的审计监督，纠正和查处各种违规行为。

　　改善金融服务。积极稳妥推进农村信用合作社管理体制和产权制度改革。进一步完善政府、银行和企业联席会议制度，支持金融部门加强管理监督，大力扶持地方中小企业发展，实现银企互动互惠。积极探索建立市场化的中小企业信贷担保机构，解决企业融资难问题。严厉打击逃废金融债务不法行为，防范和化解地方金融风险。

　　八、进一步深化各项改革，激发发展活力

　　加大民营经济发展扶持力度。加强对民营企业的指导和扶持，鼓励民营企业开展制度、技术、管理创新。抓好民营经济服务体系建设，推动建立行业组织和技术创新中心，着力解决制约民营企业发展的突出问题。积极组织民营企业参加各种国际性博览会、专业展销会，办好第四届中国（阳江）国际刀剪博览会，推进民营企业与国内外中小企业的交流与合作，提升民营经济发展水平。

　　深化国有企业改革。加快市直国有企业的改制步伐，努力完成阳江港开发总公司、阳阳铁路公司、市汽运集团、市物资总公司、市城建集团、八甲电厂等国有企业的改制工作。积极引导民资外资参与国有企业的改组改造，发展混合型和股份制经济。规范国有企业改制重组、产权交易行为。建立健全科学的考核、分配制度，强化国有资产监管，确保国有资产保值增值。稳步推进粮食流通体制改革。

　　加快投资体制改革。全面推进企业投资项目核准制和备案登记制，确立企业投资主体地位。建立新型投资体制，加强投资宏观调控和服务，及时发布产业政策、行业规划、投资信息，促进企业投资。改革投资管理，凡国家法律法规没有明确禁止的领域都允许各种经济成分的企业参与投资，积极引导社会各种资金投向基础设施和城市公用事业。

　　完善市场体系建设。大力发展资本、技术、土地、劳动力等要素市场，规范发展产权交易市场，培育发展各类社会中介组织、行业协会，逐步健全社会服务体系。继续整顿和规范市场经济秩序，深入开展食品药品安全和保护知识产权专项行动，加强对放开市场调节商品的价格监督和粮食流通市场监督，严厉打击制售假冒伪劣商品、商业欺诈和哄抬价格、价格垄断以及传销等不法行为。建立信用监督和失信惩戒制度，推进社会信用建设。

　　九、加快文化名城建设步伐，统筹推进社会事业发展

　　优先发展教育事业。深化教育管理体制和办学体制改革，落实以县为主的教育管理体制，引导非义务教育向改革找出路、向市场要资金，实行更加灵活、开放、多样的办学体制和办学模式。整合教育资源，优化学校布局，高质量普及九年义务教育，突出抓好初中教育，大力发展普通高中和中等职业教育，扩大高等教育。重点推进阳江一中、阳春一中、同心中学、英才学校、市技工学校、阳东二中等学校的扩建、迁建和新建工作。重视发展农村、山区教育事业。实施骨干示范中学建设规划，推进市职一中现代化骨干学校建设。加快基础教育课程改革，全面实施素质教育，加强德育教育，提高教育质量。加快中小学人事制度改革，提高教师队伍的师德水平和业务水平。加强社会继续教育和职业培训，构建终身教育体系。

　　大力推进科技创新。继续实施科技创新9项计划。扶持有条件的企业组建国家和省级工程技术研发中心，加快建立完善以企业为主体的技术创新体系。积极实施各类科技计划项目，推进高新技术和先进适用技术的推广应用。建设市五金刀剪等重点行业技术创新中心和服务平台，提升重点行业的科技含量和竞争力。继续实施企业信息化工程。全面铺开专业镇技术创新工作，努力新增一批省级专业镇，促进专业镇支柱产业的形成。完善华南理工大学阳江工业技术研究院建设，举办全国高等院校、科研院所科技成果（五金）展示洽谈会，推进企业与高等院校、科研机构的协作，加速科技成果转化。深化科技体制改革，完善科技服务体系。加强农村及社会发展领域的科技创新和服务工作。做好知识产权保护工作。加强全民科普教育，扎实推进国民经济和社会信息化。

　　抓好人才强市工作。实施人才 “五大工程”，培养各类高素质人才。加快粤西区域性人才市场建设，推动区域人才交流与合作，吸引优秀人才到阳江创业。健全人才选拔任用、考核评价和激励机制，营造有利于人才创业、工作、生活的社会环境。

　　加快发展文化事业。以“南海一号”沉船打捞和建设广东海上丝绸之路博物馆为契机，大力挖掘历史文化资源，深入开展乡土历史名人研究，发展具有地方特色和优势的文化产业，打造漠阳文化品牌。探索建立文化产品生产经营新机制，推进文化与经济的融合。加快图书馆、博物馆、科技馆和群众艺术馆等文化设施建设步伐，加强基层文化设施建设。鼓励民间资本参与文化建设，促进形成多元化投资、全方位发展的文化产业体系。积极开展群众性文化活动，鼓励文艺创作，繁荣文学艺术，争创“中国楹联之市”。强化文化市场监管，规范文化市场秩序。

　　增强可持续发展能力。重视做好人口和计划生育工作。抓好计生宣传教育年活动。进一步完善以农村计划生育家庭奖励扶助制度为主要内容的计划生育利益导向机制、层级动态管理责任制，加强计划生育服务网络建设，强化计划生育管理。严厉打击非医学需要的胎儿性别鉴定和以性别选择为目的的非法终止妊娠行为，综合治理人口出生性别比偏高问题。加强资源、环境保护工作。坚持开发与节约并举，推进资源节约和综合利用，突出抓好节煤、节电、节油、节水和降低重要原材料消耗工作。推广“三废”综合利用、再生资源回收利用等新技术、新设备，大力发展循环经济，逐步构筑节约型产业，建设节约型社会。推行清洁生产，坚决不上治理不达标的污染项目，加快市稀土厂搬迁工作，严格控制污染源。坚定不移地执行最严格的耕地保护制度，节约土地资源。加强生态保护和建设，维护生态安全。依法保护和合理开发利用海洋、森林等资源，重视做好漠阳江水资源和沿海地区海滩、红树林的保护工作，强化林地、林木、野生动植物和湿地资源的保护与管理。加快实施生态公益林工程，培育森林资源。推进林业生态县创建工作。依法查处无序开发利用资源等严重破坏生态行为，建设“绿色阳江”。

　　发展各项社会事业。完善疾病预防控制、卫生监督执法和医疗救治三大公共卫生体系，健全突发性公共卫生事件应急处理机制。积极开展爱国卫生运动，加强对非典、禽流感、艾滋病、结核病等的预防控制。加强农村卫生工作，完善农村卫生服务网络，改善农村医疗卫生条件。积极推进新型农村合作医疗制度。深化城镇医药卫生体制改革，加快社区卫生服务体系建设。规范医疗机构药品招标采购管理，加快医疗服务价格改革，加强卫生监督执法，提高医疗服务水平。广泛开展全民健身运动。加强体育设施、组织、活动和管理四个体系建设，加快体育产业化进程。做好民族宗教工作。加快发展妇女儿童事业，切实维护妇女儿童合法权益。加强妇幼保健工作，营造适应少年儿童健康成长环境。做好老龄工作，发展残疾人事业。完善社会救助体系。巩固和深化殡葬改革。加强全民国防教育，做好双拥共建和优抚安置工作。加强地震监测预报、灾害预防和应急救援三大体系建设，提高防震减灾综合水平。进一步做好统计、民政、外事、侨务、人防、打私、气象、档案、市志、文史等工作。

　　十、加强精神文明和民主法制建设，构建团结和谐平安社会

　　提高公民思想道德素质。坚持用邓小平理论和“三个代表”重要思想武装群众。深入开展“爱国、守法、诚信、知礼”和“二四六八公德行”等现代公民教育活动，引导全社会形成热爱祖国、遵纪守法、诚实守信、知书达理的精神风貌和行为规范，培养权利与义务相统一的现代公民意识，树立诚信、责任与谅解、宽容相统一的社会新风。深入开展文明城市、文明行业、文明单位、文明社区、文明家庭等群众性精神文明创建活动，引导城乡居民形成科学、健康、文明的生活方式，提高城乡文明程度。

　　加强民主制度建设。坚持向人大及其常委会报告工作和向人民政协通报情况制度。认真执行人大及其常委会的各项决定、决议，自觉接受人大的法律监督和工作监督。加强与人民政协的联系，支持政协履行政治协商、民主监督和参政议政职能。切实办好人大议案、代表建议和政协提案。主动听取民主党派、工商联、无党派人士和各人民团体的意见和建议。接受新闻舆论和公众监督，加强审计监督。提高决策的科学化、民主化水平，推进依法行政、科学行政、民主行政。抓好基层民主政治建设，深化政务、厂务、村务公开工作，建立社会公示和听证会制度，扩大群众对社会事务的知情权、参与权、监督权。进一步做好人民调解、矛盾调处和法律宣传等工作，不断提高公民的法律素质和政府的依法行政水平。

　　重视做好信访工作。健全信访问题及群体性事件联席会议制度，加强信访督办，着力解决好农村征地、城镇房屋拆迁、国企改革、山林纠纷等引致的上访问题，预防和妥善处置群体性事件。

　　重拳整治城乡社会治安。坚持“打防结合、预防为主，专群结合、依靠群众”方针，加强公安队伍建设，完善社会治安综合治理工作机制和城乡治安防控体系，提高防控能力。集中打压黑恶势力犯罪、严重暴力犯罪和 “双抢”、“两盗”等多发性犯罪，严厉打击“六合彩”、非法传销等违法犯罪活动，维护正常社会秩序，确保人民生命财产安全。积极有效扫除“黄赌毒”，取缔各类邪教组织。重视做好刑释解教人员安置帮教和社区矫正工作，促进社会和谐稳定。

　　切实加强安全生产。推进安全生产监管体制和执法队伍建设，落实责任制，严格许可制度，加强专项整治，强化执法监督，排查整改事故隐患。增加安全设施投入，确保安全生产形势稳定。

　　十一、坚持以人为本，切实维护群众利益

　　坚持城乡统筹，扩大就业和再就业。强化就业和再就业工作目标责任制考核。加快完善劳动力市场和基层劳动保障所建设，构建完善的就业服务体系。落实再就业小额担保贷款、岗位补贴等扶持政策，实施“零就业”家庭就业援助制度和农村贫困户就业扶贫行动，促进下岗失业人员等困难群体再就业。加强职业技能培训，提高劳动者就业能力、创业能力和职业转换能力。继续抓好高校毕业生、城镇新增劳动力就业，重视做好农村富余劳动力转移就业和复员转业军人安置工作。加强劳动保障监察和劳动关系协调工作，维护劳动者合法权益。

　　完善社保体系，提高保障水平。研究制定灵活就业人员参保办法，推动非公有制企业参保工作。加强多层次的养老保险体系建设，逐步做实养老保险个人账户，推进实施企业年金制度。进一步完善医疗保险，逐步建立社会医疗救助制度，健全多层次的医疗保障体系。完善失业保险、工伤保险和生育保险。健全和完善社会保险市级统筹制度，强化基金征缴和监管，探索基金保值增值的途径和办法，增强保障能力。积极研究失地农民养老保障问题，探索农村养老保险形式。健全和完善城乡最低生活保障制度，对符合保障标准的城乡居民做到应保尽保。

　　关心群众生产生活，维护群众利益。继续推进“十项民心工程”。加快农村贫困户泥砖住房改造和饮水工程建设步伐。建立健全城乡低收入困难群众社会救助体系，切实解决低收入群体子女入学难、看病难、打官司难和住房难等问题。大力整治群众关心的教育、医疗、药品、煤气、化肥等热点价费问题。加强和完善农村集体征地补偿款分配和使用的监管，认真查处土地承包纠纷案件和侵犯农民合法权益的违法行为。继续开展清理拖欠征地补偿款和建设领域拖欠工程款、农民工工资工作。妥善解决农村“五保户”和特困户生活困难。完善优抚保障制度，妥善解决优抚对象、企业军转干部和伤残军人的生活保障问题。

　　十二、鉴往筹来，科学编制“十一五”规划

　　用改革的精神、创新的思路、科学的方法做好规划编制工作，以科学发展观统领经济社会发展全局，坚持以人为本，按照“五个统筹”的总体要求，以及突出重点，有所为、有所不为的方针，认真分析研究“十一五”期间面临的重大战略性问题，科学界定规划编制领域，突出做好重大项目建设、经济结构调整、增长方式转变、体制改革等关系全局、带动作用强的重点领域的规划，着力解决经济社会中的突出问题。在规划的制定中，要统筹兼顾，确保“十一五”规划与各专项规划、区域规划等的协调。要广泛听取社会各界意见，增强规划工作的透明度和公众参与度，提高规划的科学化和民主化水平，真正成为体现“五个统筹”、推进机制创新、促进经济社会全面协调可持续发展的规划。

努力提高政府行政能力

　　按照建设廉洁务实高效政府、法治政府和人民满意政府的总体要求，切实加强政府自身建设，不断增强全体公务员的整体素质，努力提高政府行政能力。

　　转变政府职能。进一步深化行政管理体制改革，真正将政府职能转变到经济调节、市场监管、社会管理和公共服务上来。认真贯彻落实行政许可法和《全面推进依法行政实施纲要》，切实加强政府法制建设，严格按照法定权限和程序行使权力、履行职责。深化行政审批制度改革，简化和规范行政许可程序，强化许可行为监督。继续清理行政审批事项，进一步减少、简化审批程序和手续，明确审批时限，实现依法行政和高效行政的统一。

　　改进机关作风。牢固树立科学发展观和正确政绩观，弘扬求真务实精神，大兴调查研究之风，力戒官僚主义、文牍主义、形式主义，做到察实情、说实话、务实事、求实效。把狠抓工作落实作为转变工作作风的关键环节，进一步完善决策目标、执行责任、考核监督“三个体系” ，努力形成“深、实、细、严、快”的工作作风，切实把各项工作落到实处、抓出实效、干出实绩。

　　健全督办考核制度。创新行政监督机制，改进和完善政绩考核办法，探索建立政府绩效评估体系和经济社会发展评价体系，提高行政监督效能，确保政令畅通。落实工作责任制，把各项工作逐项分解，明确责任，加大督查力度，特别是对市委、市政府决定的事项和事关全局、难度较大的工作，要进行重点督办，推动各项工作的落实。

　　加强廉政建设，提高公务员素质。结合开展保持共产党员先进性和“理想、责任、能力、形象”教育活动，进一步加强公务员队伍建设，完善公务员选人用人机制和考核指标体系，强化公务员培训，推进公务员轮岗交流，努力提高公务员队伍特别是领导干部科学判断形势、总揽全局、应对复杂局面、领导经济工作、解决实际问题的能力。加大依法行政力度，规范行政行为。落实党风廉政建设责任制，推进领导干部廉洁自律。坚决纠正损害群众利益的不正之风，依法查处违法案件，使公务员队伍始终保持勤政廉政、谦虚谨慎、不骄不躁、艰苦奋斗的作风，不断开创政府工作新局面。

　　各位代表，时代赋予我们神圣使命，我们的任务艰巨而光荣。让我们高举邓小平理论和“三个代表”重要思想伟大旗帜，紧密团结在以胡锦涛同志为总书记的党中央周围，在市委的正确领导下，全面落实科学发展观，解放思想，与时俱进，开拓创新，扎实工作，为把阳江建设成为工业新城、生态绿城、旅游美城、文化名城和现代化海滨城市而努力奋斗！
